# *In vitro* interspecies interactions between methicillin-resistant *Staphylococcus aureus* and *Pseudomonas aeruginosa*: effects on bacterial growth, antibiotic susceptibility, and transcriptomic reprogramming

**DOI:** 10.3389/fcimb.2026.1845513

**Published:** 2026-06-12

**Authors:** Xinliang Peng, Hang Zhou, Wanchang Liu, Maohui Li, Zhongyuan He, Hang Liu, Xingyu Yang, Zhaoyang Guo, Wentao Zhang, Wei Fu, Hanchao Liang, Lei Shi, Rui Deng, Yongjun Dang, Chao Xie, Yunying Wang, Zhongliang Deng, Youliang Ren, Lei Chu

**Affiliations:** 1 Department of Orthopaedics, the Second Affiliated Hospital of Chongqing Medical University, Chongqing, China; 2 Department of Laboratory Medicine, Guizhou Provincial People’s Hospital, Guiyang, China; 3 Department of Orthopaedics, Guizhou Provincial People’s Hospital, Guiyang, China; 4 Basic Medicine Research and Innovation Center for Novel Target and Therapeutic Intervention, Ministry of Education, College of Pharmacy, The Second Affiliated Hospital of Chongqing Medical University, Chongqing Medical University, Chongqing, China; 5 Department of Orthopaedics, Center for Musculoskeletal Research, University of Rochester Medical Center, Rochester, NY, United States; 6 Department of Laboratory Medicine, the Second Affiliated Hospital of Chongqing Medical University, Chongqing, China

**Keywords:** antibiotic susceptibility, *in vitro* co-culture, methicillin-resistant staphylococcus aureus, polymicrobial infections, pseudomonas aeruginosa, transcriptional reprogramming

## Abstract

**Introduction:**

Polymicrobial bone infections (PBIs) are associated with greater clinical severity and present more significant therapeutic challenges than monomicrobial infections. Methicillin-resistant *Staphylococcus aureus* (MRSA) and *Pseudomonas aeruginosa* (PA) are the most frequently isolated pathogens in PBIs. However, despite previous studies have suggested both competitive and cooperative interactions between these two pathogens, their underlying molecular mechanisms and the impact on antibiotic susceptibility remain incompletely understood.

**Methods:**

Under *in vitro* co-culture conditions, bacterial growth and biofilm formation of MRSA and PA were evaluated by colony-forming unit (CFU) enumeration, crystal violet staining, and live/dead fluorescence staining. Minimum inhibitory concentrations (MICs) of clinically relevant antibiotics were determined for each species following co-culture to evaluate reciprocal alterations in antibiotic susceptibility. Additionally, RNA sequencing (RNA-seq) was performed on both species separately after co-culture to profile transcriptomic changes underlying their polymicrobial interaction.

**Results:**

At an MRSA: PA ratio of 1:1, MRSA CFU remained close to the initial inoculum throughout the experiment, with maximum CFU reaching only 1% of that in monoculture. MRSA biofilm biomass in the presence of PA cell-free supernatant was reduced by 33% at 36 hours. Notably, antibiotic susceptibility testing showed that MRSA exhibited increased MIC values for levofloxacin and daptomycin, with MICs 1.53 ± 0.00-fold and 2.00 ± 0.00-fold higher, respectively, than those in the monoculture group. Meanwhile, PA showed increased MIC values to colistin, with a 2.00 ± 0.00-fold higher MIC, but reduced MIC values for levofloxacin and ceftazidime, both showing a 0.50 ± 0.00-fold lower MIC compared with the monoculture group. Transcriptomic analysis of MRSA revealed significant increases in gene expression related to ribosome biogenesis, oxidative phosphorylation, and stress response pathways. PA co-cultured with MRSA downregulated multidrug-resistant efflux pump genes and upregulated pore-forming protein genes.

**Conclusion:**

PA inhibited MRSA growth *in vitro* co-culture at a 1:1 ratio, accompanied by altered antibiotic susceptibility profiles and transcriptional reprogramming. This study provides mechanistic insights into MRSA-PA interactions and establishes an experimental framework for developing targeted therapies against PBIs.

## 1 Introduction

Post-traumatic osteomyelitis (PTOM) is a common and clinically challenging complication following fractures or severe traumatic injuries (Lew and Waldvogel, 2004). Its pathogenesis is multifactorial, involving extensive bone destruction, compromised local blood supply, and dysregulated host immune responses (Geng et al., 2025). Clinically, PTOM is associated with substantial morbidity and mortality, while also imposing a significant economic burden on healthcare systems (Flores et al., 2024). Current standard-of-care strategies rely primarily on aggressive surgical debridement combined with prolonged high-dose antibiotic therapy (Depypere et al., 2020; Reinhart et al., 2024). Nevertheless, even under standardized treatment protocols, PTOM remains difficult to eradicate, with high recurrence rates and unsatisfactory cure outcomes, underscoring the urgent need for novel therapeutic approaches, particularly in the context of polymicrobial infections.

Accumulating epidemiological evidence indicates that polymicrobial infections constitute a major etiological factor in PTOM (Ren et al., 2023). Approximately 47.9% of PTOM cases are caused by infections involving two or more microbial species (Depypere et al., 2022; Jorge et al., 2018; Metsemakers et al., 2018). Compared with monomicrobial infections, polymicrobial infections are typically associated with more severe clinical manifestations, increased treatment complexity, and reduced responsiveness to antibiotic therapy.

Among the causative pathogens, *Staphylococcus aureus* is the most prevalent Gram-positive bacterium implicated in PTOM. This organism exhibits strong virulence potential, inducing robust inflammatory responses and extensive tissue damage, while readily establishing chronic infections. In particular, methicillin-resistant *Staphylococcus aureus* (MRSA) poses a substantial therapeutic challenge due to its resistance to methicillin and other β-lactam antibiotics (Mapar et al., 2025; Song et al., 2025). The pathogenic persistence of MRSA has been attributed to multiple mechanisms, including biofilm formation, expression of antibiotic resistance determinants, and the emergence of small colony variants (SCVs), which are characterized by slow growth and reduced metabolic activity (Ogunware and Kielian, 2025; Schilcher and Horswill, 2020).

Among Gram-negative pathogens, *Pseudomonas aeruginosa* (PA) is another major contributor to orthopedic and implant-associated infections. PA produces a wide array of virulence factors, such as elastase and pyocyanin, which promote tissue destruction and facilitate immune evasion. Clinical studies have reported a co-occurrence rate of MRSA and PA in PTOM cases of up to 16.6% (Peng et al., 2017), suggesting that these two pathogens frequently coexist within the same infectious niche and may engage in complex interspecies interactions.

Notably, emerging evidence suggests that the coexistence of MRSA and PA may lead to altered bacterial growth behaviors and modulated antibiotic responsiveness under co-culture conditions, implying that interspecies interactions can profoundly influence bacterial physiology beyond simple coexistence (Aunon et al., 2025; Keim et al., 2024). Such phenotypic adaptations raise the possibility that underlying transcriptional reprogramming may be triggered in response to polymicrobial environments. However, the molecular basis of these interaction-driven adaptations and their implications for antibiotic susceptibility remain largely unexplored (Landa et al., 2024; Trizna et al., 2020).

To address these knowledge gaps, we performed transcriptomic analysis combined with antibiotic susceptibility testing to evaluate MRSA-PA interactions under co-culture conditions. We hypothesize that interspecies interactions will trigger adaptive transcriptional reprogramming in MRSA and PA, significantly altering their antibiotic susceptibility profiles. Through this study, we aim to establish an experimental framework for understanding polymicrobial interactions in PTOM and to inform the development of targeted therapeutic strategies.

## 2 Materials and methods

### 2.1 Bacterial strains and culture conditions

Methicillin-resistant *Staphylococcus aureus* (MRSA, ATCC BAA-1717) and *Pseudomonas aeruginosa* (PA, ATCC 15692) were obtained from Baosai Biotechnology. Luria–Bertani (LB) broth (Solarbio, China), LB agar (Solarbio, China), mannitol salt agar (MSA, Hopebio, China), and *Pseudomonas* CN agar (CA, HuanKai Microbial, China) were used for routine bacterial culture and selective isolation. Both MRSA and PA were cultured under standard aerobic conditions at 37 °C. Although their optimal nutrient requirements are not identical, LB broth provided a compatible, non-selective condition that supported the growth of both organisms and enabled the assessment of interspecies interactions under standardized conditions (Cendra et al., 2019; Wijesinghe et al., 2019). For direct-contact co-culture experiments, bacterial cultures were incubated at 37 °C with shaking at 200 rpm. For indirect-contact co-culture experiments, cultures were incubated statically at 37 °C.

### 2.2 Co-culture of MRSA and PA and growth curve measurement

To standardize the initial inoculum sizes, isolated colonies of MRSA and PA were separately inoculated into 5 mL of LB broth and cultured overnight at 37 °C with shaking at 200 rpm (Fu et al., 2025). Based on growth-curve analysis, both organisms had reached stationary phase before co-culture (**Supplementary Figure 3**). The overnight cultures were then adjusted according to species-specific OD600–CFU/mL calibration curves and further verified by serial dilution and CFU enumeration to obtain the required initial inoculum densities. Bacterial suspensions were adjusted to approximately 1 × 10^7^ CFU/mL and further serially diluted to obtain suspensions of 1 × 10^6^ and 1 × 10^5^ CFU/mL.

To monitor bacterial growth dynamics under co-culture conditions over a 48-h period, two complementary co-culture systems were employed: direct-contact co-culture under shaking conditions and indirect-contact co-culture using a Transwell system (Briaud et al., 2019; Lafuente-Gracia et al., 2021).

For direct-contact co-culture, MRSA and PA were mixed at three initial ratios based on viable bacterial cell numbers, expressed as CFU/mL (MRSA: PA = 1:1, 1:100, and 100:1). Specifically, MRSA and PA suspensions were prepared separately and adjusted to the required CFU/mL according to species-specific OD600–CFU/mL calibration curves. Equal volumes (2.5 mL) of the adjusted bacterial suspensions were then combined to obtain the desired MRSA: PA ratios and cultured at 37 °C with shaking at 200 rpm. And species-specific bacterial quantification during co-culture was performed using selective plate. MRSA was enumerated on MSA, whereas PA was enumerated on CA. LB agar was used as a non-selective reference medium. The selectivity of these media was validated using MRSA monoculture, PA monoculture, and MRSA-PA mixed-culture suspensions plated in parallel on LB agar, mannitol salt agar, and cetrimide agar. All CFU experiments were performed with three independent biological replicates. For each biological replicate, selected dilutions were plated in triplicate as technical replicates.

Indirect-contact co-culture was performed using 0.4 μm polycarbonate Transwell inserts, which allow diffusion of soluble factors while preventing the passage of intact bacterial cells. To verify the absence of detectable bacterial migration between chambers, aliquots were collected separately from the upper and lower chambers at designated time points and plated onto species-selective media (**Supplementary Figure 1**). MRSA was initially added to the lower chamber, while PA was added to the upper chamber, using the same three initial ratios (MRSA: PA = 1:1, 1:100, and 100:1) as described above. To maintain equal culture volumes, fresh LB broth was added as needed to the lower chamber. In parallel experiments, the positions of MRSA and PA in the upper and lower chambers were exchanged. Plates were sealed with a transparent hydrophobic membrane to minimize evaporation and incubated at 37 °C. At the indicated time points (0–48 hours), bacterial suspensions from the lower chamber were collected, and growth was monitored by measuring OD_600_ using a microplate reader (Alford et al., 2022).

### 2.3 Antimicrobial susceptibility testing of co-cultured bacteria

To assess the impact of co-culture on antibiotic susceptibility, MRSA and PA were co-cultured under direct-contact conditions for 24 hours and 48 hours, with corresponding monocultures used as controls. Following co-culture, mixed bacterial suspensions were plated onto MSA and CA for selective isolation of MRSA and *P. aeruginosa*, respectively. Well-isolated colonies (3–5 per condition) were randomly selected and resuspended in sterile saline, and bacterial suspensions were adjusted to a 0.5 McFarland standard (Trizna et al., 2020).

Antibiotic susceptibility testing was performed using the VITEK 2 automated system (bioMérieux, France) with GP and GN susceptibility cards, following the manufacturer’s instructions. Quality control was performed using reference strains *S. aureus* ATCC 29213 and *P. aeruginosa* ATCC 27853. Susceptibility results were interpreted based on Clinical and Laboratory Standards Institute (CLSI) guidelines (M100, 2021).

Antibiotics used for susceptibility testing were obtained from commercial sources and prepared according to manufacturer instructions. Clinically relevant antibiotics were tested for both bacterial groups. For the MRSA group, the antibiotics tested included benzylpenicillin (PEN), oxacillin (OXA), ceftiofur (XNL), gentamicin (GEN), levofloxacin (LVX), moxifloxacin (MXF), erythromycin (ERY), clindamycin (CLI), linezolid (LZD), daptomycin (DAP), teicoplanin (TEC), vancomycin (VAN), tigecycline (TGC), rifampin (RIF), and trimethoprim/sulfamethoxazole (SXT). For the PA group, the antibiotics tested included ticarcillin/clavulanic acid (TIM), piperacillin/tazobactam (TZP), ceftazidime (CAZ), cefepime (FEP), imipenem (IPM), meropenem (MEM), amikacin (AMK), tobramycin (TOB), ciprofloxacin (CIP), levofloxacin (LVX), and colistin (CST). Minimum inhibitory concentration (MIC) values were determined for each antibiotic and bacterial strain and compared with those of monoculture controls. For comparison across conditions, MIC values were normalized to monoculture controls by calculating the ratio of MICs obtained under co-culture and monoculture conditions. MIC assays were performed with three independent biological replicates using freshly prepared bacterial inocula for each replicate.

### 2.4 Cell-free supernatants of MRSA and PA

To prepare cell-free supernatants (CFS), MRSA and PA were cultured overnight at 37 °C with shaking at 200 rpm. Bacterial cultures were centrifuged at 6,000 × g for 10 min, and supernatants were collected and passed through 0.22 µm syringe-driven filters to remove residual bacterial cells (Vilela et al., 2015). Sterility was confirmed by plating 100 µL of filtered supernatant onto LB agar and incubating at 37 °C for 24 h. No bacterial colonies were detected after incubation, confirming the absence of viable bacteria in the filtered CFS (**Supplementary Figure 4**). Filtered supernatants were aliquoted and stored at −20 °C until further use.

### 2.5 Impact of bacterial supernatants on biofilm formation by MRSA and PA

To assess the effects of cell-free supernatants (CFS) on biofilm formation, MRSA and PA were exposed to heterologous bacterial supernatants. Briefly, in 24-well plates, 100 µL of MRSA suspension was mixed with 900 µL of either LB/PBS (1:1, v/v) or LB/PA CFS (1:1, v/v). Similarly, 100 µL of PA suspension was combined with 900 µL of either LB/PBS (1:1, v/v) or LB/MRSA CFS (1:1, v/v). Cultures were incubated at 37 °C under static conditions to allow biofilm formation (Zhang et al., 2021a).

After incubation, planktonic cells were removed, and wells were gently washed three times with PBS and air-dried for 1 h. Biofilms were stained with 0.1% (w/v) crystal violet solution. Following staining, crystal violet was solubilized using 1 mL of absolute ethanol, and absorbance at 600 nm was measured using a microplate reader for biofilm quantification (Andersen et al., 2024). Biofilm formation assays were performed with three independent biological replicates, and each biological replicate included three technical replicate wells for each condition.

To assess biofilm viability, live/dead staining was performed using a bacterial viability staining kit (Solarbio). Biofilms were cultured in confocal dishes and stained according to manufacturer instructions. Fluorescence images were acquired using a laser scanning confocal microscope (Olympus). NucGreen stains total bacterial cells, whereas EthD-III selectively labels bacteria with compromised membranes (Blanco-Cabra et al., 2022). Image processing and three-dimensional reconstruction were performed using OlyVIA (v3.3) and Imaris (v9.0.1), and fluorescence signal quantification was conducted using ImageJ (Zhang et al., 2021b).

### 2.6 Transcriptomic analysis of co-cultured bacteria

To investigate the responses of MRSA and PA during their interaction, we compared the gene expression profiles of the two bacteria after 24 hours and 48 hours of co-culture, as well as monoculture. The procedure was as follows: total RNA was extracted using the TRIzol method and quality control was performed using the Agilent 2100 Bioanalyzer. rRNA was then removed using the Ribo-Zero kit to enrich mRNA. Next, strand-specific cDNA libraries were constructed using the NEBNext^®^ Ultra™ II kit and sequenced on the Illumina NovaSeq™ platform. For bioinformatics analysis, differential expression analysis was performed using DESeq2 (threshold set at |log2Fold Change| > 0.585 and P < 0.05), followed by GO, KEGG, and GSEA enrichment analysis of significantly differentially expressed genes.

### 2.7 RT-qPCR validation of selected differentially expressed genes

Selected representative differentially expressed genes identified by RNA-seq were validated by RT-qPCR. For MRSA, *qoxA*, *qoxB*, and *ctaA* were analyzed, with *gyrB* used as the internal reference gene. For PA, *phzB2*,*pqsE* were analyzed, with *rpsL* used as the internal reference gene. Relative gene expression was calculated using the 2−ΔΔCq method. The primer sequences used for RT-qPCR are listed in **Supplementary Table 3**.

## 3 Results

### 3.1 Validation of selective media for quantifying MRSA and PA in co-culture

**Figure 1:**
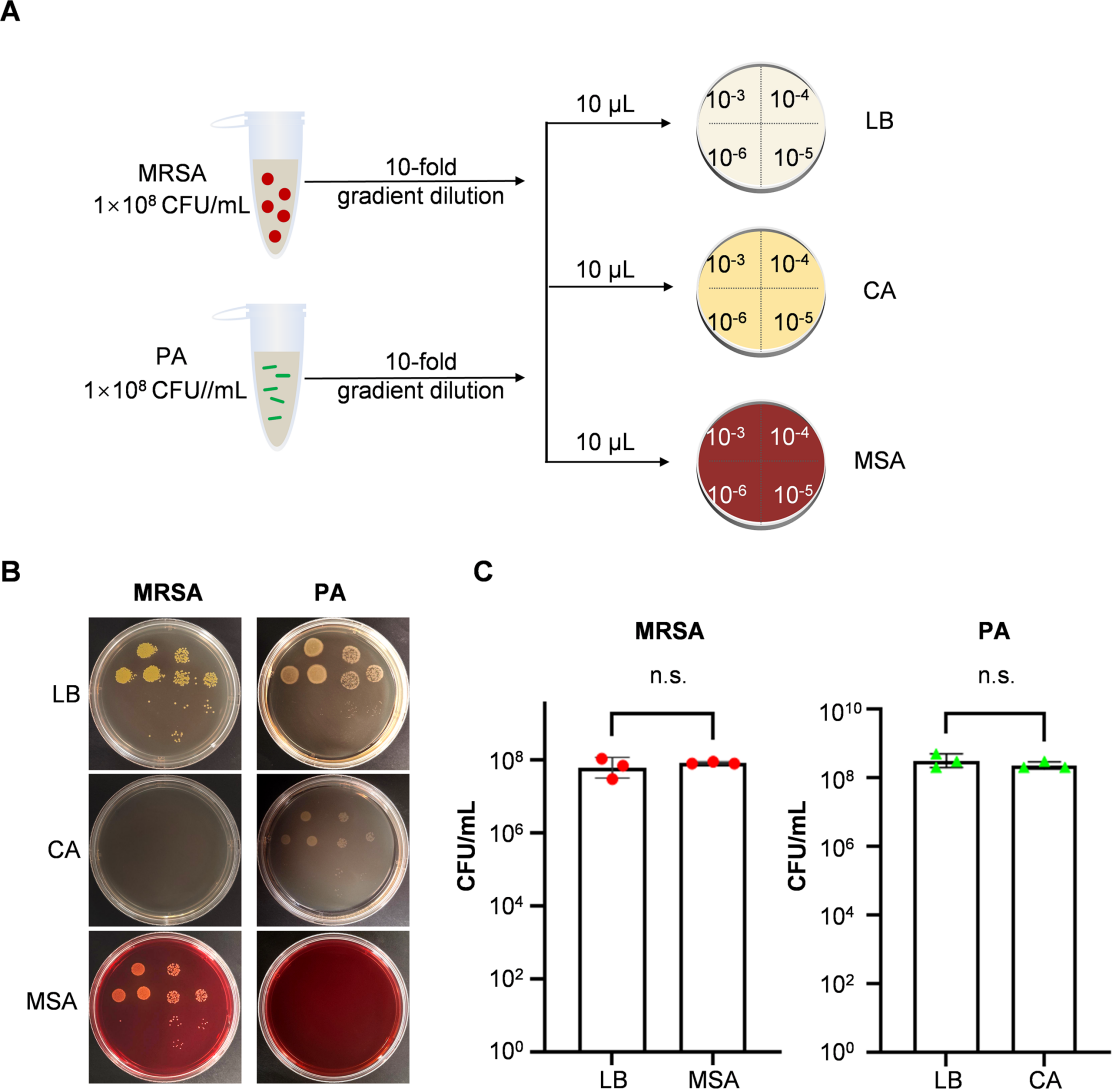
Validation of selective media for enumeration MRSA and PA. **(A)** Suspensions of MRSA and PA (1 × 10^8^ CFU/mL) were serially 10-fold diluted from 10^-3^ to 10^-6^, and 10 μL of each dilution was plated onto LB agar, CA, and MSA, with each dilution tested in triplicate. **(B)** Following incubation at 37 °C for 24 hours, CA selectively supported PA growth, whereas MSA selectively supported MRSA growth. Colony counts and morphologies were comparable to those observed on LB agar. **(C)** Quantification analysis of bacterial CFU at the 10^-5^ dilution (n = 3). Data are shown as mean ± s.d.; n.s., not significant.

To enable accurate quantification of MRSA and PA under co-culture conditions, we first evaluated the selectivity of CA and MSA. Serial dilutions of MRSA and PA monocultures were plated onto LB agar, CA, and MSA, respectively (**Figure 1A**). Following incubation, CA selectively supported the growth of PA, whereas MSA selectively supported the growth of MRSA, with colony morphology and counts comparable to those observed on non-selective LB agar (**Figure 1B**). Quantitative analysis at the 10^-5^ dilution confirmed that CFU enumeration on selective media did not significantly differ from that on LB agar (**Figure 1C**), validating the use of CA and MSA for reliable species-specific quantification in subsequent co-culture experiments.

### 3.2 PA inhibits MRSA growth in co-culture

**Figure 2:**
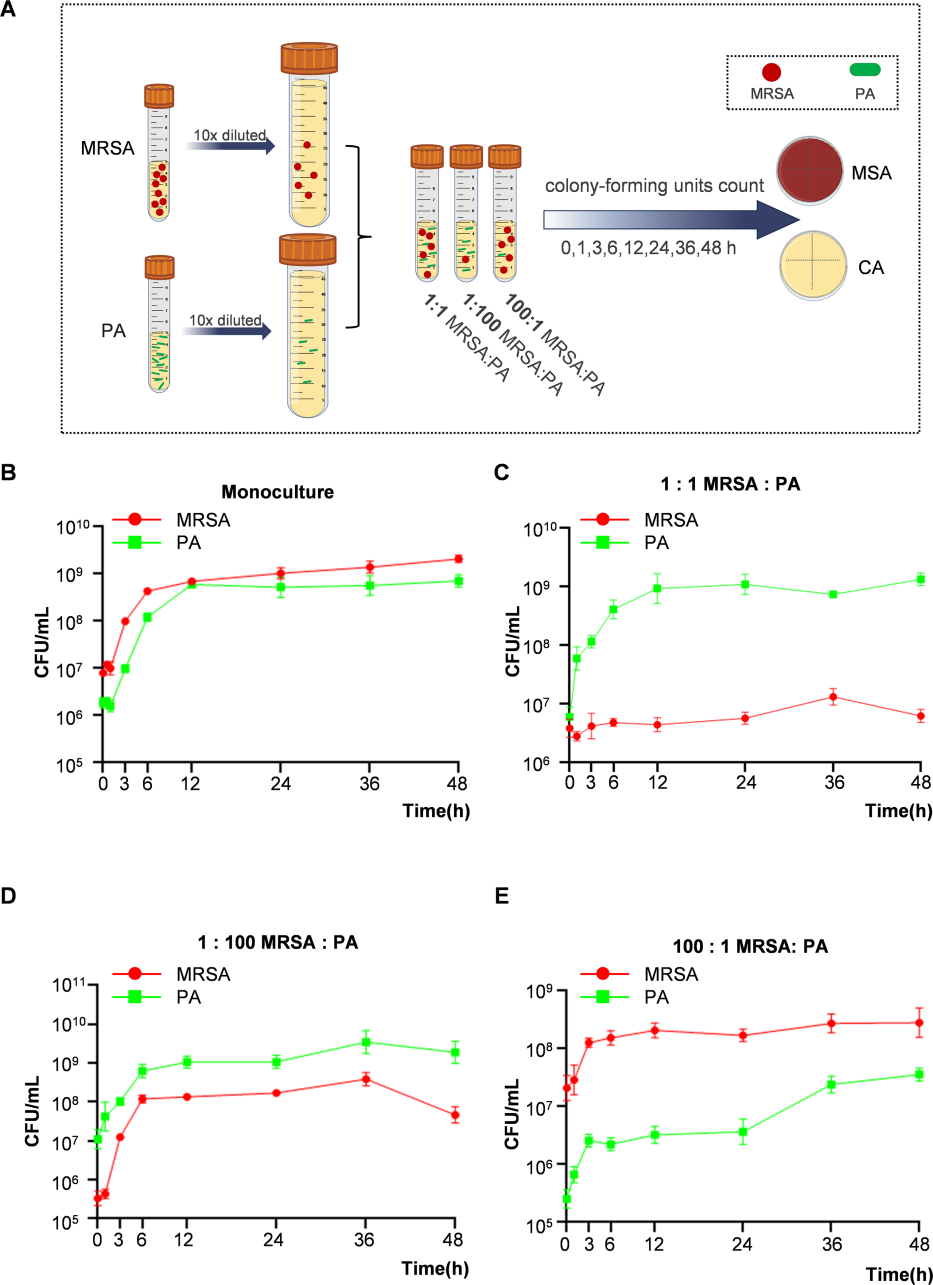
Growth dynamics of MRSA and PA under direct-contact co-culture. **(A)** Schematic overview of the direct-contact co-culture experimental design. Overnight cultures of MRSA and PA were adjusted to approximately 10^7^ CFU/mL and mixed at initial MRSA:PA ratios of 1:1, 1:100, and 100:1. Cultures were incubated at 37 °C with shaking at 200 rpm, and samples were collected at designated time points (0-48 hours) for CFU enumeration. **(B)** Growth curves of MRSA and PA in monoculture. **(C–E)** Growth curves of MRSA and PA at initial MRSA:PA ratios of 1:1 **(C)**, 1:100 **(D)**, and 100:1 **(E)**.

In the *in vitro* direct-contact co-culture model, MRSA and PA exhibited significant competitive interactions, with bacterial growth highly dependent on the initial inoculum ratio. At an initial MRSA: PA ratio of 1:1, PA growth closely resembled that observed in monoculture, whereas MRSA outgrowth was markedly restricted and remained close to the initial inoculum level throughout the observation period (**Figures 2B, C**). The maximum MRSA count in the 1:1 co-culture was 7.12 ± 0.14 log10 CFU/mL, compared with 9.32 ± 0.08 log10 CFU/mL in MRSA monoculture. After back-transformation of log10 CFU/mL values, this corresponded to approximately 0.6% of the monoculture maximum. However, the MRSA count at 48 h was 6.79 log10 CFU/mL, which remained slightly above the initial inoculum level of 6.58 log10 CFU/mL. When the initial MRSA number was disadvantageous (1:100), PA growth remained unaffected, while MRSA showed limited proliferation, reaching a maximum of approximately 10^8^ CFU/mL, which was lower than its monoculture level (**Figures 2B, D**). In contrast, when MRSA was in absolute abundance (100:1), its growth kinetics were comparable to monoculture, while PA growth was markedly restricted and failed to reach monoculture levels (**Figures 2B, E**). A comprehensive comparison revealed that PA dominated the *in vitro* competitive environment. Interestingly, when PA abundance was fixed (**Figures 2C, D**), the low-density MRSA group (1:100) exhibited better growth adaptability than the high-density MRSA group (1:1), suggesting that high-density MRSA might trigger a more potent inhibitory mechanism by PA or adversely affect its own microenvironment.

To quantitatively characterize the growth patterns observed in **Figure 2**, kinetic parameters including the maximal specific growth rate (μmax), lag phase duration, and maximal population density (CFUmax) were extracted from CFU-based growth curves and are summarized in **Table 1**. At an initial MRSA: PA ratio of 1:1, MRSA exhibited a marked reduction in μmax accompanied by a pronounced extension of the lag phase (25.0 ± 19.1 h), consistent with severe growth suppression. When MRSA was initially outnumbered by PA (1:100), μmax partially recovered and the lag phase returned to monoculture levels, although CFUmax remained reduced. In contrast, PA maintained relatively stable growth kinetics across most co-culture conditions, with moderate reductions in μmax and CFUmax only when introduced at a numerical disadvantage (100:1).

**Table 1:** Growth kinetic parameters of MRSA and PA under direct-contact monoculture and co-culture conditions.

Species	Condition	Initial ratio (MRSA:PA)	μmax (log_10_CFU/h)	Lag time (h)	CFUmax (log_10_CFU/mL)
MRSA	Monoculture	–	0.50 ± 0.09	3.0 ± 0.0	9.32 ± 0.08
MRSA	Co-culture	1:1	0.13 ± 0.10	25.0 ± 19.1	7.12 ± 0.14
MRSA	Co-culture	1:100	0.73 ± 0.03	3.0 ± 0.0	8.59 ± 0.17
MRSA	Co-culture	100:1	0.40 ± 0.04	2.3 ± 1.2	8.51 ± 0.20
PA	Monoculture	–	0.42 ± 0.06	3.0 ± 0.0	8.92 ± 0.03
PA	Co-culture	1:1	1.00 ± 0.05	1.0 ± 0.0	9.19 ± 0.08
PA	Co-culture	1:100	0.59 ± 0.29	1.0 ± 0.0	9.62 ± 0.23
PA	Co-culture	100:1	0.50 ± 0.14	1.7 ± 1.2	7.56 ± 0.11

Growth kinetic parameters were estimated from CFU-based growth curves obtained under direct-contact mono- and co-culture conditions. Data are presented as mean ± s.d. from three independent experiments.

**Figure 3:**
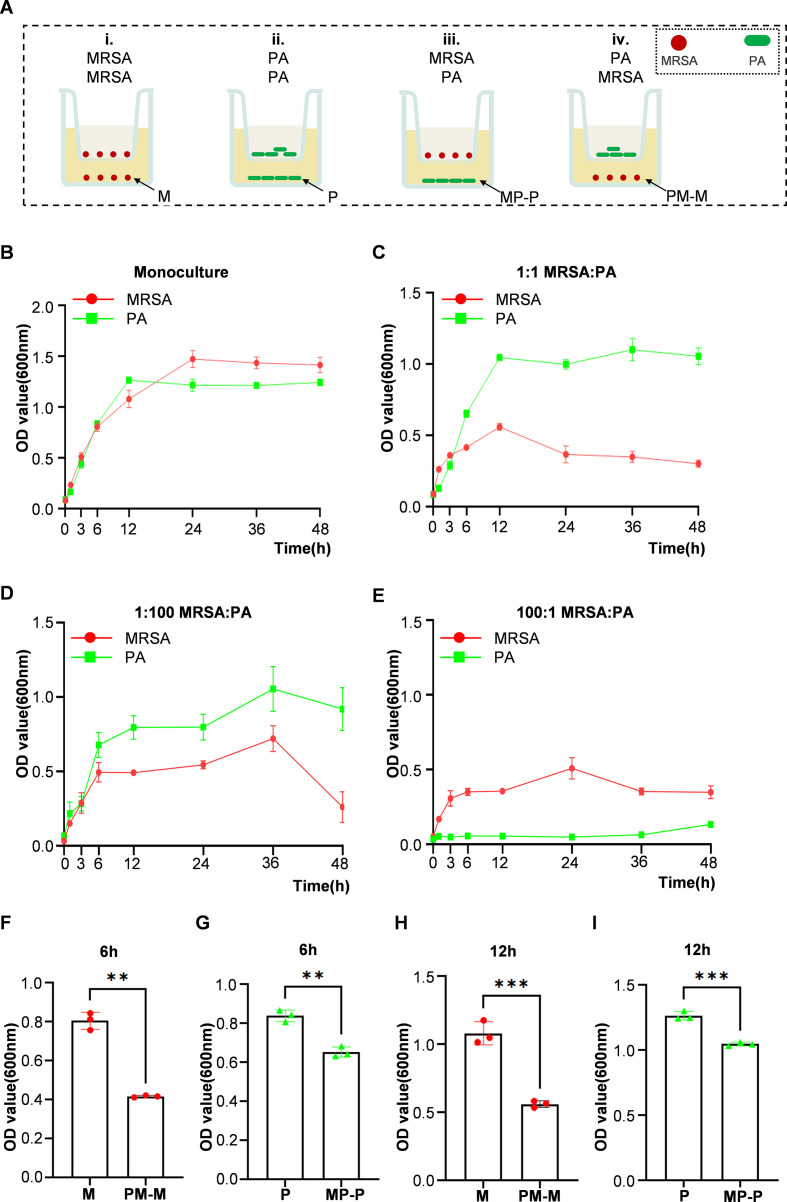
MRSA and PA were co-cultured in indirect-contact. **(A)** An overview of the method employed to establish indirect-contact co-culture between the two bacterial strains. i: Both upper and lower chambers were inoculated with MRSA. ii: Both upper and lower chambers were inoculated with PA. iii: The upper chamber was inoculated with MRSA, and the lower chamber was inoculated with PA. iv: The upper chamber was inoculated with PA, and the lower chamber was inoculated with PA. Bacterial suspensions from the lower chamber were collected for OD_600_ measurement, with the groups represented as M, P, MP-P, and PM-M, respectively. **(B)** Growth curves of MRSA and PA in monoculture. **(C–E)** Growth curves of MRSA and PA at initial MRSA:PA ratios of 1:1 **(C)**, 1:100 **(D)**, and 100:1 **(E)**. **(F–I)** Quantitative comparison of MRSA and PA growth at 6 hours **(F, G)** and 12 hours **(H, I)** under monoculture and indirect co-culture conditions at an initial MRSA:PA ratio of 1:1. Data are presented as mean ± s.d. (n = 3). Statistical significance was determined using an unpaired t-test. **P < 0.01; ***P < 0.001.

In the *in vitro* indirect-contact co-culture model, a Transwell system was used to physically separate the two bacteria, allowing only the exchange of secreted molecules (**Figure 3A**). Bacterial concentration was assessed by measuring the optical density (OD) of the lower chamber bacterial suspension at 600 nm. The results indicated that the bacterial growth trends in each experimental group were consistent with those observed in the direct-contact co-culture model (**Figures 3B–E**), suggesting that PA inhibition of MRSA primarily depends on secreted soluble factors rather than direct cell-to-cell contact. Furthermore, this inhibitory effect displayed a clear density-dependent characteristic.

### 3.3 Antibiotic susceptibility testing of co-cultured bacteria

**Figure 4:**
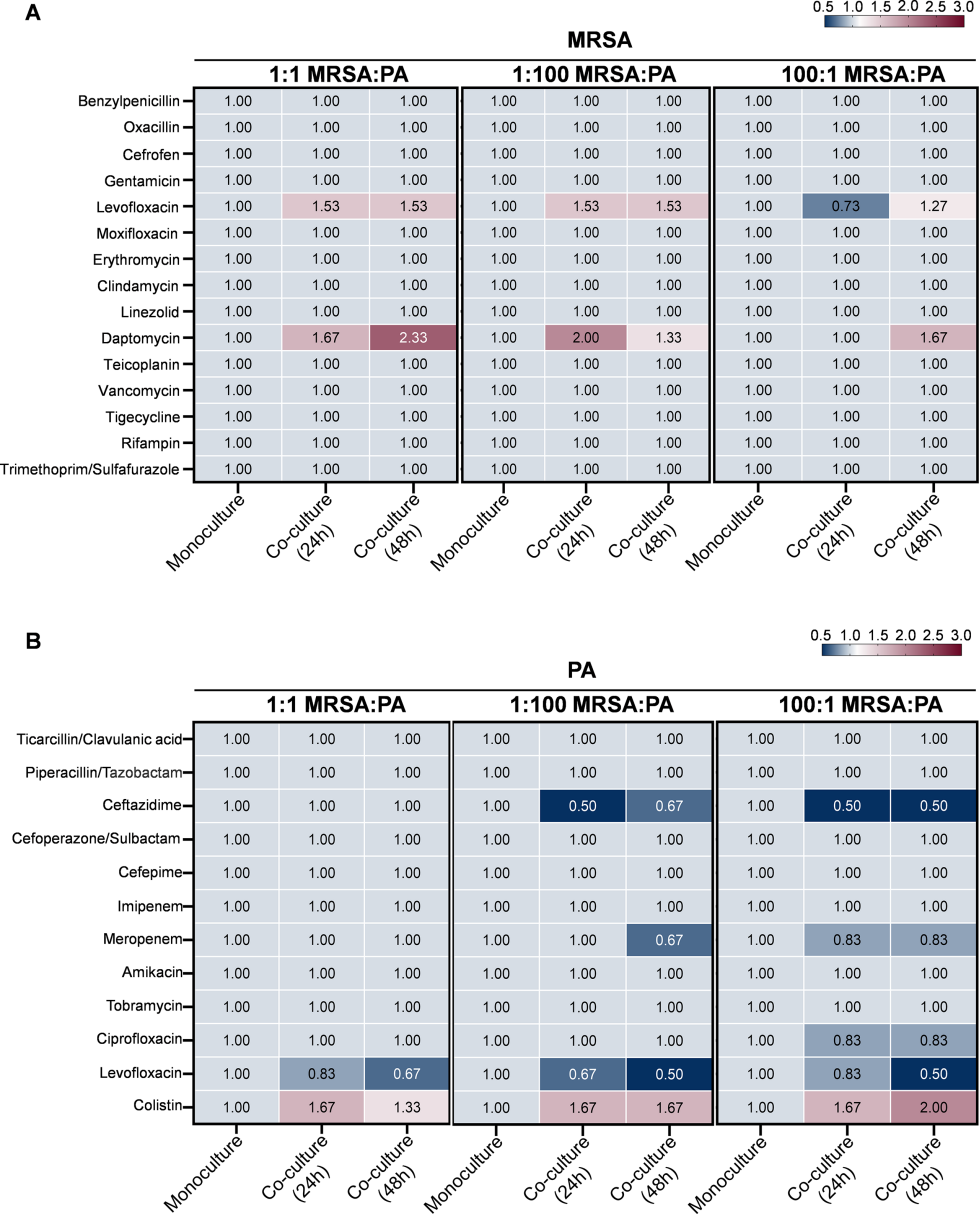
Antibiotic susceptibility profiles of MRSA and PA following co-culture. **(A)** Antibiotic susceptibility results of monoculture MRSA and MRSA strains isolated after 24 hours and 48 hours of co-culture with PA. **(B)** Antibiotic susceptibility results of monoculture PA and PA strains isolated after 24 hours and 48 hours of co-culture with MRSA. MIC values are expressed as fold changes relative to the corresponding monoculture controls. Values >1 indicate increased antibiotic tolerance, whereas values <1 indicate increased antibiotic sensitivity. Data are presented as mean ± s.d. (n = 3).

To assess whether interspecies interactions alter antibiotic susceptibility, the minimum inhibitory concentrations (MICs) of MRSA and PA to a panel of clinically relevant antibiotics were determined following co-culture and normalized to monoculture controls (**Figure 4**).

Specifically, when MRSA and PA were co-cultured at a 1:1 ratio, MRSA showed increased MIC values to LVX, with MICs elevated by 1.53 ± 0.00-fold at both 24 hours and 48 hours relative to monoculture. DAP MIC values also increased, with MICs elevated by 1.67 ± 0.47-fold at 24 hours and further increasing to 2.33 ± 1.24-fold at 48 hours. In contrast, when MRSA and PA was 1:100, LVX MIC ratios remained comparable to that observed under 1:1 co-culture conditions (1.53 ± 0.00-fold), whereas DAP MIC values was significantly increased (2.00 ± 0.00-fold at 24 hours, *P* < 0.05). When MRSA dominated (100:1), LVX MIC values increased, as indicated by an increase in MIC to 1.27 ± 0.37-fold at 48 hours relative to monoculture, whereas DAP MIC values remained largely unchanged (1.67 ± 0.47-fold at 48 hours) (**Figure 4A**).

For PA, in the 1:1 co-culture, PA showed reduced MIC values for LVX, with MICs reduced to 0.83 ± 0.23-fold at 24 hours and 0.67 ± 0.23-fold at 48 hours, but showed increased MIC values for CST, with MICs elevated by 1.67 ± 0.47-fold at 24 hours and 1.33 ± 0.47-fold at 48 hours. When PA dominated the co-culture (1:100), reduced MIC values for CAZ (0.50 ± 0.00-fold at 24 hours, *P* < 0.05), MEM (0.67 ± 0.23-fold at 48 hours), and LVX (0.67 ± 0.23-fold at 24 hours and 0.50 ± 0.00-fold at 48 hours, *P* < 0.05 at 48 hours) was observed, while CST MIC values remained unchanged (1.67 ± 0.47-fold). In contrast, when PA was present at lower relative abundance (100:1), susceptibility to most antibiotics was largely maintained, including CAZ (0.50 ± 0.00-fold at both 24 hours and 48 hours) and LVX (0.83 ± 0.23-fold at 24 hours and 0.50 ± 0.00-fold at 48 hours, *P* < 0.05 at 48 hours). However, a modest increase in CST MIC was detected at 48 hours (2.00 ± 0.00-fold, *P* < 0.05) (**Figure 4B**).

### 3.4 PA supernatant suppresses MRSA biofilm formation

**Figure 5:**
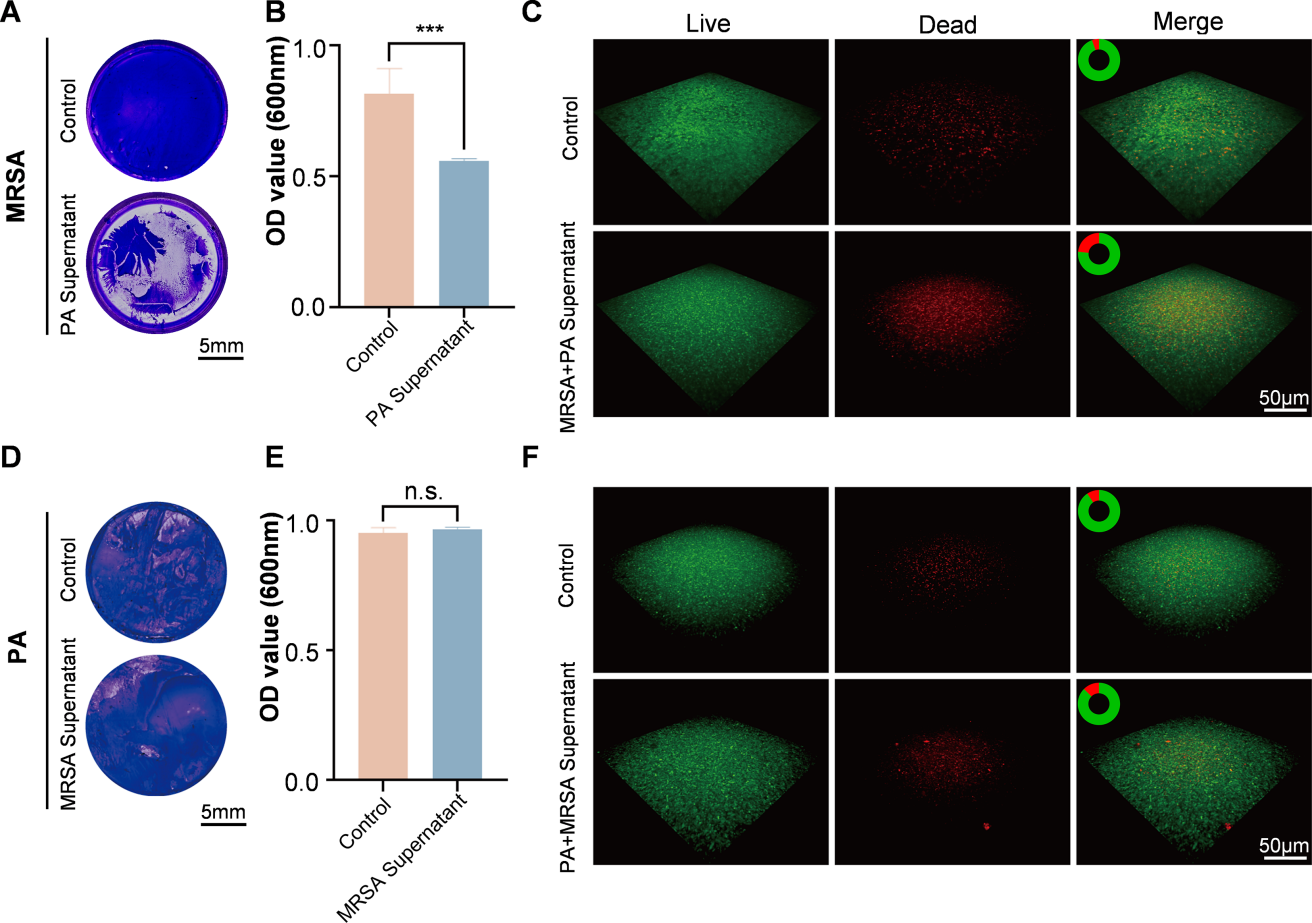
Effects of MRSA and PA supernatants on biofilm formation by MRSA and PA. **(A)** Crystal violet staining of biofilm formed after MRSA was incubated in PA supernatant for 36 hours. Scale bar = 5 mm. **(B) **Biofilm quantification of MRSA after incubation in PA supernatant. The biofilm was dissolved using absolute ethanol, and the absorbance was measured at OD_600_. n=3. **(C)** Fluorescent live/dead staining of MRSA biofilms. **(D)** Crystal violet staining of biofilm formed after PA was incubated in MRSA supernatant for 36 hours. Scale bar = 5 mm. **(E)** Biofilm quantification of PA after incubation in MRSA supernatant. **(F)** Fluorescent live/dead staining of PA biofilms. Live bacteria are labeled green, and dead bacteria are labeled red. The live/dead bacteria ratio is represented in doughnut charts. Scale bar = 50μm. ***P < 0.001; n.s., not significant (*P* ≥ 0.05).

We investigated the effects of MRSA and PA CFS on biofilm formation using crystal violet staining and quantitative analysis. Our results showed that PA CFS significantly inhibited MRSA biofilm formation (**Figures 5A, B**), whereas MRSA CFS did not affect PA biofilm formation compared to the control group (**Figures 5D, E**). Additionally, we performed live/dead bacterial staining using the EX3000 kit (Solarbio), which revealed a reduction in live MRSA cells after PA CFS treatment (**Figure 5C**). Live bacteria were stained green by NucGreen, while dead bacteria appeared red due to EthD-III staining.

### 3.5 Transcriptional regulation of MRSA post co-cultured with PA

**Figure 6:**
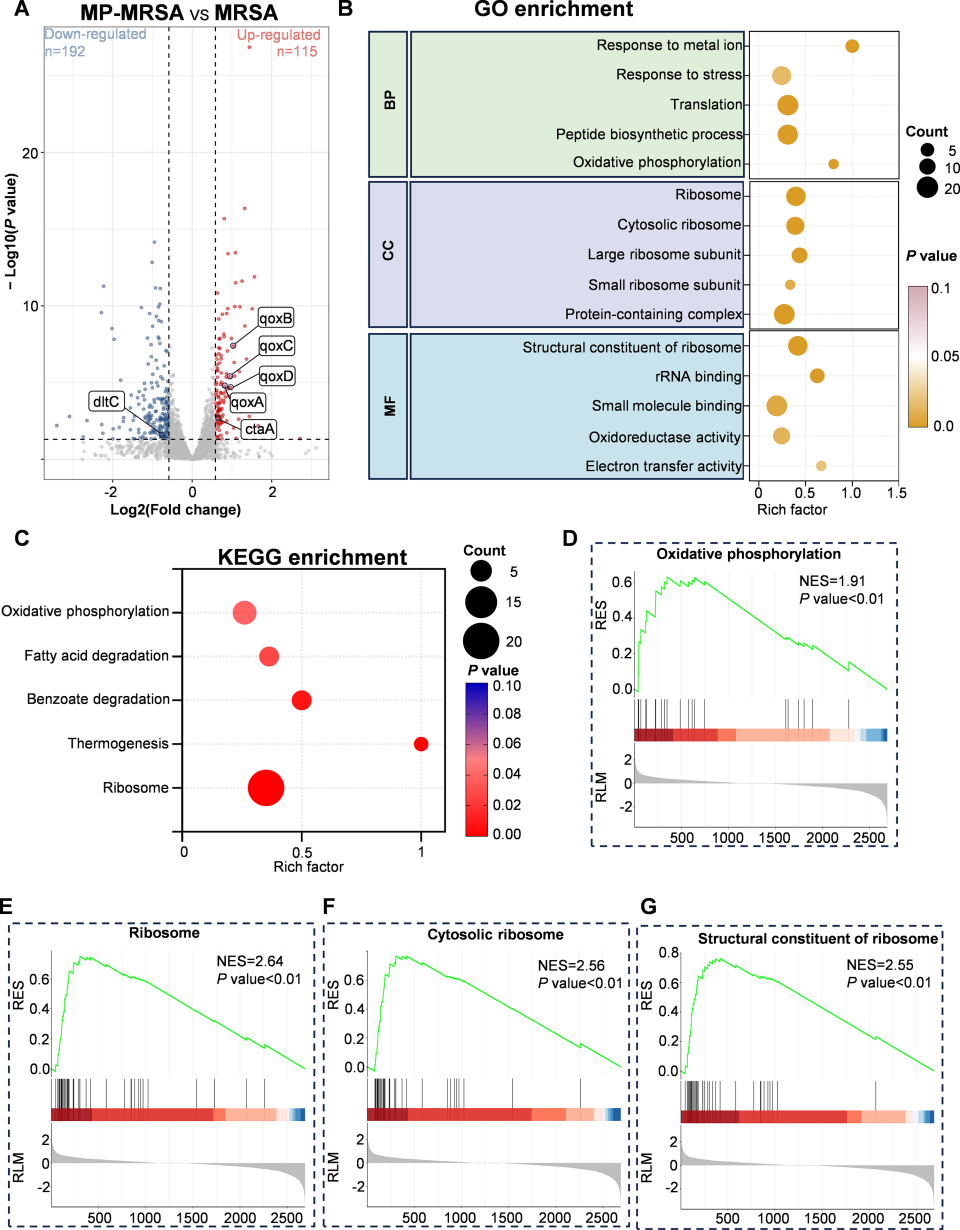
Transcriptomic alterations of MRSA in response to co-culture conditions. **(A)** Volcano plot of differentially expressed genes. Blue points represent significantly downregulated genes, while red points represent significantly upregulated genes (*P*-value < 0.05 and |log2FoldChange| > 0.585). **(B)** Gene Ontology (GO) enrichment analysis of differentially expressed genes, categorized into Biological Process (BP), Cellular Component (CC), and Molecular Function (MF). **(C)** Kyoto Encyclopedia of Genes and Genomes (KEGG) enrichment analysis of differentially expressed genes. **(D–G)** Gene Set Enrichment Analysis (GSEA) of differentially expressed genes.

Compared to the MRSA group, 307 significantly differentially expressed genes (DEGs) were identified in MRSA co-cultured with PA, of which 115 were upregulated and 192 were downregulated (**Figure 6A**). Gene Ontology (GO) enrichment analysis revealed that the DEGs were significantly enriched in Cellular Components (CC), particularly ribosomes (including cytosolic ribosomes, large ribosomal subunits, and small ribosomal subunits) and protein complexes. In terms of Biological Processes (BP), these genes were predominantly involved in key pathways such as metal ion response, translation, peptide biosynthesis, oxidative phosphorylation, and stress response. At the Molecular Function (MF) level, the DEGs were significantly enriched in categories such as ribosomal structure composition, rRNA binding, small molecule binding, oxidoreductase activity, and electron transfer activity (**Figure 6B**). Kyoto Encyclopedia of Genes and Genomes (KEGG) enrichment analysis further highlighted ribosome biogenesis as the most significantly enriched pathway. Additionally, pathways related to energy metabolism and material utilization were enriched, including oxidative phosphorylation, fatty acid degradation, and benzoate degradation (**Figure 6C**). Gene Set Enrichment Analysis (GSEA) further confirmed the upregulation of these pathways, with significant enrichment in oxidative phosphorylation, ribosome, cytosolic ribosome and structural constituent of ribosome, as indicated by the normalized enrichment scores (NES) of 1.91 (*P* < 0.01), 2.64 (*P* < 0.01), 2.56 (*P* < 0.01) and 2.55 (*P* < 0.01), respectively (**Figures 6D–G**).

### 3.6 Transcriptional regulation of PA post co-cultured with MRSA

**Figure 7:**
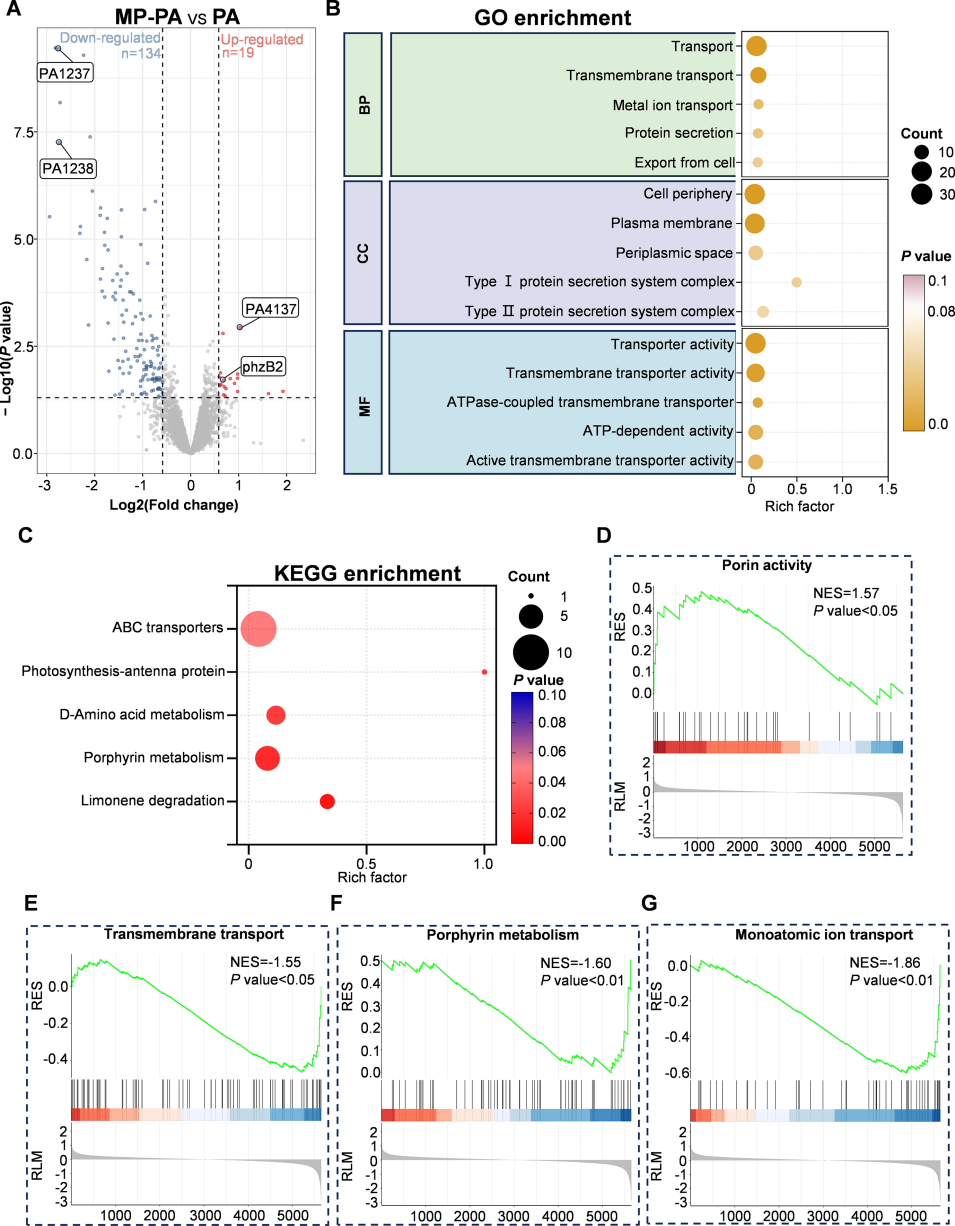
Transcriptomic alterations of PA in response to co-culture conditions. **(A)** Volcano plot of differentially expressed genes. Blue points represent significantly downregulated genes, while red points represent significantly upregulated genes (*P*-value < 0.05 and |log2FoldChange| > 0.585). **(B)** Gene Ontology (GO) enrichment analysis of differentially expressed genes, categorized into Biological Process (BP), Cellular Component (CC), and Molecular Function (MF). **(C)** Kyoto Encyclopedia of Genes and Genomes (KEGG) enrichment analysis of differentially expressed genes. **(D–G)** Gene Set Enrichment Analysis (GSEA) of differentially expressed genes.

Compared to the PA group, 153 significantly differentially expressed genes (DEGs) were identified in PA co-cultured with MRSA, with 19 upregulated and 134 downregulated genes (**Figure 7A**). GO analysis revealed significant enrichment of the DEGs in several functional categories. Specifically, CC analysis highlighted significant alterations in the extracellular region, plasma membrane, periplasmic space, and Type I and Type II protein secretion system complexes. The significantly enriched BP predominantly involved processes related to transmembrane transport, such as transport, metal ion transport, protein secretion, and cellular excretion. At the MF level, the DEGs were notably enriched in categories such as transporter activity, transmembrane transporter activity, ATPase-coupled transmembrane transporter activity, and active transmembrane transporter activity (**Figure 7B**). KEGG enrichment analysis highlighted limonene degradation, porphyrin metabolism, and D-amino acid metabolism as the most significantly enriched pathway. Furthermore, ABC transporter pathways were also enriched (**Figure 7C**). GSEA analysis revealed that porin activity had a NES of 1.57 (*P* < 0.05). In contrast, transmembrane transport (NES = -1.55, *P* < 0.05), porphyrin metabolism (NES = -1.60, *P* < 0.01), and monoatomic ion transport (NES = -1.86, *P* < 0.01) were significantly downregulated (**Figures 7D, E**).

## 4 Discussion

In this study, we investigated the complex interspecies interactions between MRSA and PA *in vitro* co-culture models. These results provide new insights into the interactions between MRSA and PA, elucidating their effects on growth dynamics, antibiotic susceptibility profiles, and biofilm formation.

We observed a significant inhibitory effect of PA on MRSA growth in the co-culture model, with the effect being highly dependent on the initial bacterial ratio (Alford et al., 2022; Pastar et al., 2013). When MRSA and PA were co-cultured at a 1:1 ratio, MRSA outgrowth was markedly restricted compared with monoculture, whereas PA growth remained largely unaffected. Although the maximum MRSA count in the 1:1 co-culture was approximately 1% of that observed in MRSA monoculture after back-transformation of log10 CFU/mL values, the MRSA count at 48 h remained slightly above the initial inoculum level. Therefore, this finding is more consistent with growth inhibition or bacteriostatic-like suppression rather than direct bactericidal activity by PA. However, when MRSA predominated at a 100:1 ratio, its growth was nearly unaffected, suggesting a density-dependent competitive interaction. This finding aligns with previous studies showing that PA can outcompete other bacteria via quorum sensing (QS) mechanisms, which regulate the production of virulence factors (Smith et al., 2017). At higher cell densities, QS enhances the production of these factors, leading to stronger inhibition of competing bacteria like MRSA. Although our study observed this competitive interaction, we did not provide direct evidence to confirm that QS mechanisms, such as the Las and Rhl systems, are responsible for this effect. Further investigation is required to elucidate the precise role of QS and other potential factors in these dynamics (Beaudoin et al., 2017; Murray et al., 2022). Additionally, our study was limited to the 1:1, 1:100, and 100:1 ratios, which may not fully capture the broader range of bacterial densities that could influence these interactions.

Our antibiotic susceptibility testing revealed *in vitro* shifts in MIC values for both MRSA and PA under co-culture conditions. Specifically, MRSA showed an increased MIC values for LVX and DAP. For example, at the MRSA: PA 1:100 ratio, the MIC of DAP increased to 2.00 ± 0.00-fold at 24 h(*P* < 0.05), whereas the MIC of LVX was elevated by 1.53 ± 0.00-fold relative to monoculture. These changes were interpreted as tolerance-like MIC shifts rather than classical resistance. The increased MIC of LVX in co-cultured MRSA may reflect interaction-induced metabolic adaptation, as transcriptomic analysis showed enrichment of oxidative phosphorylation, ribosome biogenesis, and stress-response pathways, without clear enrichment of canonical levofloxacin resistance genes or major efflux-pump genes (Kumar et al., 2025; Vestergaard et al., 2019). These changes are well-recognized hallmarks of stress tolerance and persistence in *S. aureus*, including small colony variants and biofilm-associated phenotypes (Kaatz et al., 2006; Proctor, 2019; Proctor et al., 2014). The increased MIC of DAP may be related to broader cell-envelope or membrane-associated adaptation, because daptomycin activity is strongly influenced by bacterial membrane properties and cell-envelope physiology (Kumar et al., 2025; Lee et al., 2016; Lorusso et al., 2022; Wu et al., 2024).

Conversely, PA showed reduced MIC values for LVX, with MICs reduced to 0.83 ± 0.23-fold at 24 h and 0.67 ± 0.23-fold at 48 h, as well as to CAZ, with a 0.50 ± 0.00-fold reduction in MIC (*P* < 0.05), while displaying increased MIC values for CST, with the MIC elevated to 2.00 ± 0.00-fold (*P* < 0.05). This opposite pattern may reflect a trade-off-like adaptive response involving different antibiotic targets and resistance mechanisms. Reduced colistin susceptibility is commonly associated with lipopolysaccharide modification and altered outer-membrane charge, whereas susceptibility to levofloxacin and ceftazidime can be influenced by antibiotic influx and efflux. Consistently, PA after co-culture showed downregulation of efflux pump-associated genes, including PA1237 and PA1238, and upregulation of the porin-related gene PA4137. Together, these findings suggest that polymicrobial interactions can modulate antibiotic susceptibility in a context-dependent manner. Differences from previous studies may be related to experimental setup, strain background, antibiotic class, and whether planktonic or biofilm models were used.

We further investigated the effect of bacterial supernatants on biofilm formation, which is a critical factor in the persistence and resistance of bacterial pathogens in infections (Al-Hamoud et al., 2025; Marques et al., 2023). Our results indicated that PA supernatants significantly inhibited MRSA biofilm formation, whereas MRSA supernatants had no significant effect on PA biofilm formation. Moreover, PA-derived cell-free supernatants may contain soluble bacterial products, including metabolites, secreted factors, and signaling molecules, which could affect MRSA independently of live PA cells (Cao et al., 2020; Orazi et al., 2019). While the present study employed crystal-violet quantification and live/dead staining to assess biofilm formation, previous studies have quantified SA biofilms using a GFP-reporter strain and CFU enumeration (Radlinski et al., 2017; Trizna et al., 2020). Despite the methodological differences, both approaches support the same conclusion: PA supernatant significantly suppresses SA biofilm development as evidenced by reduced fluorescence or crystal-violet absorbance, whereas SA supernatant exerts no appreciable effect on PA biofilms (Niggli et al., 2023; Orazi et al., 2019).

Our results suggest that the MRSA–PA interaction represents a dynamic process involving PA predominance, MRSA stress adaptation, and partial coexistence rather than complete competitive exclusion. Although PA showed a competitive advantage in growth assays and PA-derived soluble products inhibited MRSA biofilm formation, MRSA was not completely eliminated from the co-culture system. These findings indicate that MRSA can persist under co-culture stress, while PA becomes relatively dominant (Deleon et al., 2014; Filkins et al., 2015; Landa et al., 2024).

Transcriptome sequencing of MRSA following co-culture with PA (versus MRSA monoculture) revealed that differentially expressed genes were significantly enriched in pathways such as ribosomal biosynthesis, oxidative phosphorylation, and stress response. This indicates that MRSA is devoting substantial resources to enhancing its protein synthesis and energy metabolism to maintain essential cellular functions. This metabolic shift explains the changes in its antibiotic sensitivity. For LVX, MRSA displayed an increasing trend in MIC after co-culture. Although no significant enrichment of traditional resistance genes or efflux pumps was observed, the upregulation of *qoxA-D*, which encode key components of the respiratory chain, together with the upregulation of *ctaA*, suggests that MRSA enhances energy conversion efficiency by synthesizing more efficient cytochrome caa_3_ (Chateau et al., 2022). This may contribute to the increased MIC values observed under competitive conditions. Notably, this restructuring of the respiratory chain likely disrupts the proton motive steady state. Since LVX uptake into cells relies on proton motive potential, its attenuation reduces LVX uptake, thereby elevating the MIC (Son et al., 2024; Zhang et al., 2026). Regarding DAP, studies have shown that upregulation of the dltC gene increases bacterial surface hydrophobicity, thereby enhancing susceptibility to the hydrophobic antibiotic DAP. This aligns with the findings in this study, where downregulation of the dltC gene resulted in elevated MIC values for DAP (Burel et al., 2021; Grein et al., 2020; Guo et al., 2025; Nugrahani et al., 2022).

Unlike the adaptive response observed in MRSA, PA exhibited adaptive changes toward energy resource optimization in co-culture. Our transcriptomic data reveal that differentially expressed genes are highly enriched in functions such as type I/II secretion systems, transmembrane transport, and ABC transporters, but their expression is predominantly downregulated. Notably, this included multiple downregulated multidrug efflux pump genes (PA1237 and PA1238), which are expected to impair the efflux capacity of PA against a range of antibiotics (Rhouma et al., 2021; Wu-Chen et al., 2023). Concurrently, upregulation of the pore protein gene PA4137 was associated with reduced MIC values for antibiotics including CAZ, MEM, CIP, and LVX, which exert their bactericidal effects intracellularly or within the periplasm. In contrast, colistin acts by targeting negatively charged lipopolysaccharides on the outer membrane, thereby disrupting membrane integrity (Kong et al., 2023; Mondol et al., 2025; Paredes-Amaya et al., 2023). The upregulation of pore protein genes is unlikely to potentiate this mechanism. It may contribute to maintaining osmotic stability in competitive environments, thereby potentially contributing to the increased CST MIC observed under co-culture conditions. Moreover, the upregulation of *phzB2*, which encodes a key virulence factor in PA, ensures its competitive advantage. This strategy aligns with our experimental findings: PA demonstrated dominance in growth competition during *in vitro* co-culture with MRSA, and its supernatant effectively inhibited MRSA biofilm formation.

Several limitations should be acknowledged. First, this study examined only three initial MRSA: PA ratios (1:1, 1:100, and 100:1), whereas bacterial proportions in clinical polymicrobial infections may be more heterogeneous and dynamic. Second, we used well-characterized reference strains to establish a reproducible MRSA and PA co-culture model; however, clinical MRSA and PA isolates, as well as other Gram-negative pathogens involved in post-traumatic osteomyelitis, such as *Escherichia coli* and *Enterobacter cloacae*, may exhibit distinct virulence, resistance, metabolic, and regulatory profiles (Depypere et al., 2022; Wang et al., 2017; Zhang et al., 2022). Third, transcriptomic analysis was performed at two time points (24 hours and 48 hours), and post-co-culture recovery assays were not conducted to determine whether the observed MIC shifts were reversible after removal from the co-culture environment. Therefore, the temporal dynamics of transcriptional changes and the stability of altered antimicrobial susceptibility remain to be further elucidated. Fourth, although LB broth provided a standardized *in vitro* condition, it does not recapitulate the bone infection microenvironment, and environmental parameters such as pH, dissolved oxygen, and nutrient availability were not directly monitored during co-culture (Deleon et al., 2014; Filkins et al., 2015). Future studies using dynamic ratio models, multiple clinical isolates, additional clinically relevant pathogens, recovery assays, and bone infection-relevant systems are warranted to further validate these findings.

To conclude, our study demonstrates that PA inhibits MRSA growth via secreted soluble factors, with reciprocal changes in antibiotic susceptibility and species-specific transcriptional reprogramming. Mechanistically, MRSA adapts to co-culture by upregulating respiratory chain-related genes *qoxA-D* and *ctaA*. This adaptation enhances energy metabolism but inadvertently increases tolerance to levofloxacin and daptomycin. In contrast, PA downregulates multiple multidrug efflux pumps while upregulating pore-forming proteins, resulting in reduced MIC values for certain β-lactams and fluoroquinolones. Notably, PA retains its competitive advantage through enhanced expression of the key virulence factor *PhzB2*. By combining transcriptomic analysis with phenotypic assays, our study establishes an experimental framework for understanding polymicrobial interactions in bone infections. Furthermore, these findings support the future exploration of rational combination therapy, anti-biofilm approaches, and emerging interventions, such as vaccines, nanomedicine-based drug delivery, or ultrasound-assisted antimicrobial strategies, for complex polymicrobial and biofilm-associated bone infections.

## Data Availability

The original contributions presented in the study are included in the article/**Supplementary Material**. Further inquiries can be directed to the corresponding authors.
