# Supplementary material for: In vitro interspecies interactions between methicillin-resistant Staphylococcus aureus and Pseudomonas aeruginosa: effects on bacterial growth, antibiotic susceptibility, and transcriptomic reprogramming
Source: Front Cell Infect Microbiol. 2026 Jun 12;16:1845513. doi: 10.3389/fcimb.2026.1845513 (PMC13303858; doi:10.3389/fcimb.2026.1845513)
Supplement: Supplementary file 1 [file DataSheet1.docx]

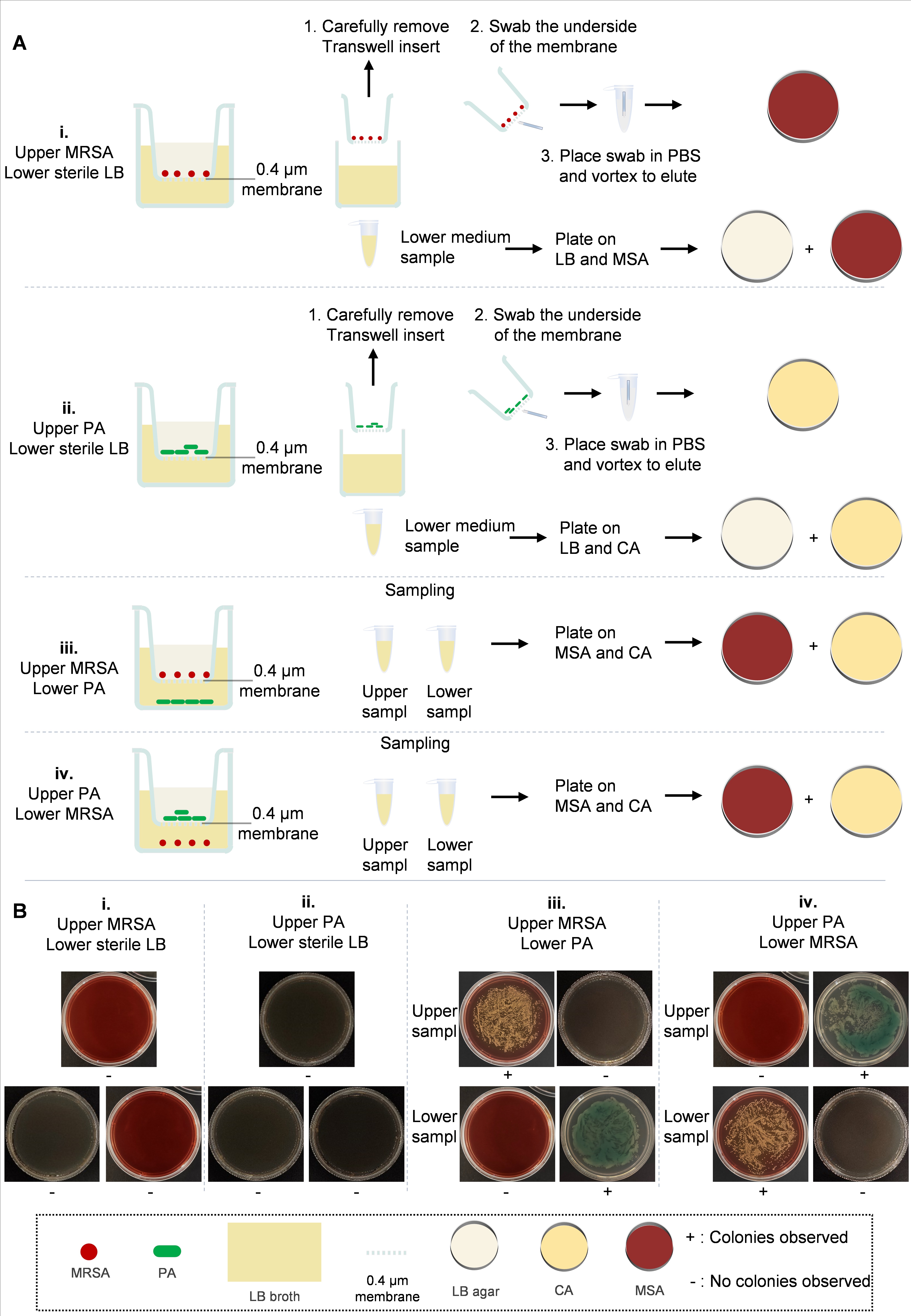


**Supplementary Figure S1. Validation of physical separation in the Transwell co-culture system.**


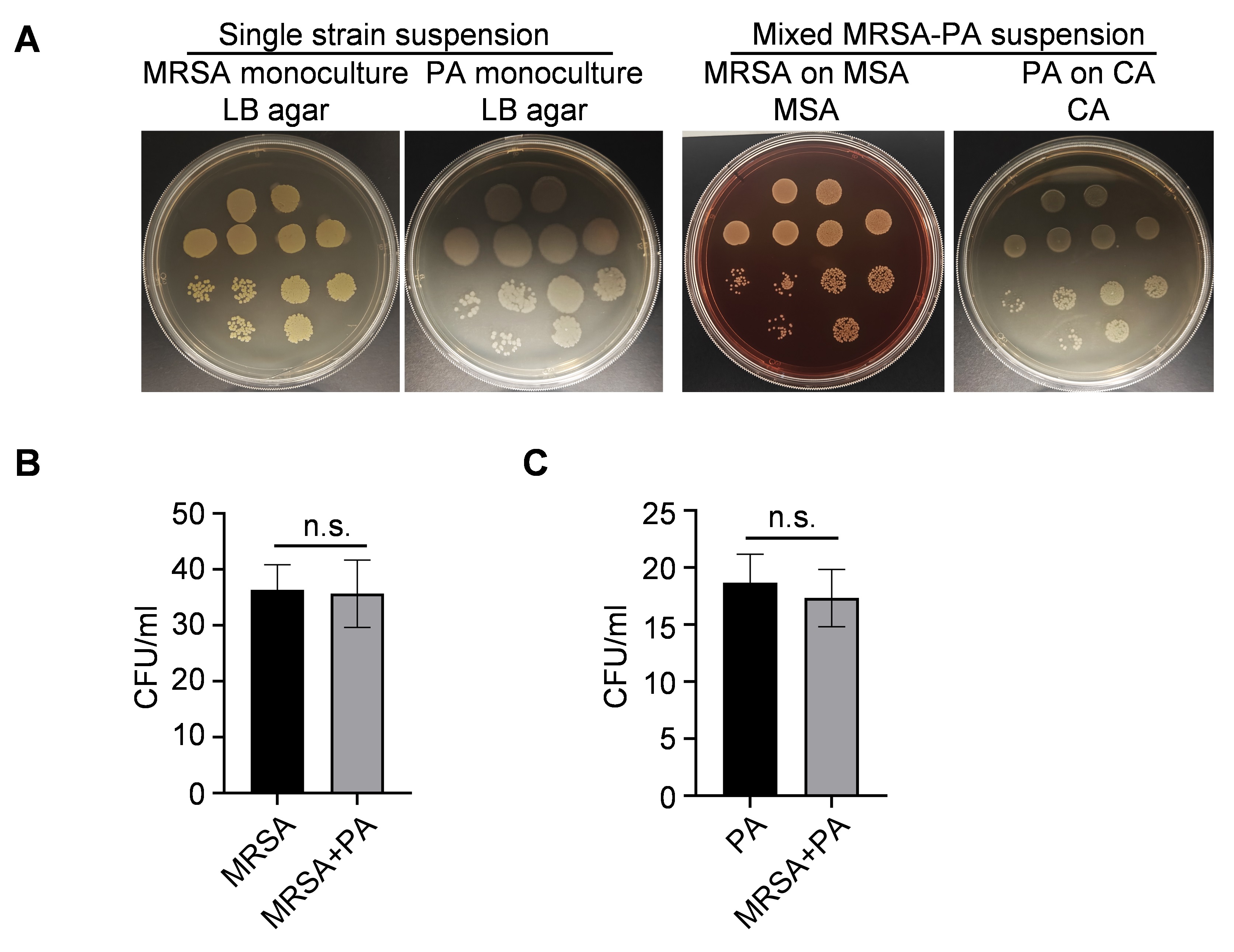


**Supplementary Figure S2. Validation of selective media for species-specific enumeration of MRSA and PA.** (A) Mixed bacterial suspensions containing both MRSA and PA were serially diluted and plated in parallel on non-selective LB agar, mannitol salt agar (MSA, selective for MRSA), and Pseudomonas CN agar (CA, selective for PA). (B) Quantitative data are presented as mean ± SD from three independent biological replicates unless otherwise indicated.


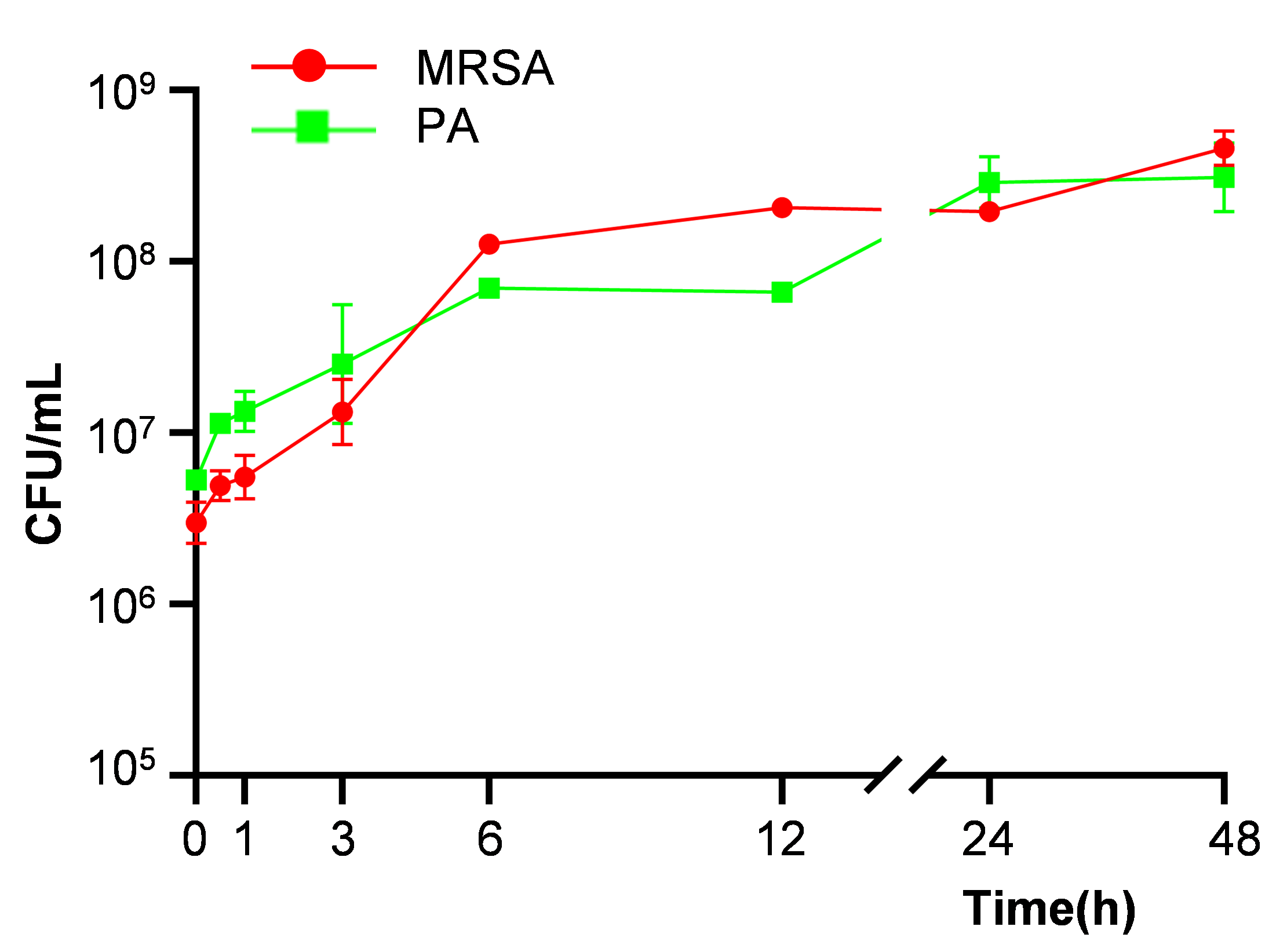


**Supplementary Figure S3. Growth curves of MRSA and PA in LB broth based on CFU enumeration.**


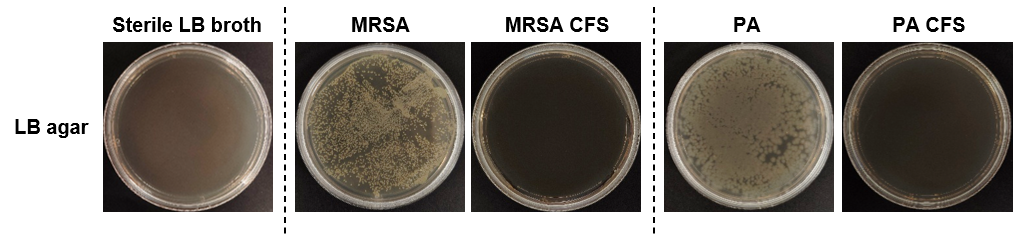


**Supplementary Figure S4. The bacterial enumeration of cell‑free supernatants from MRSA and PA on LB agar.**

s
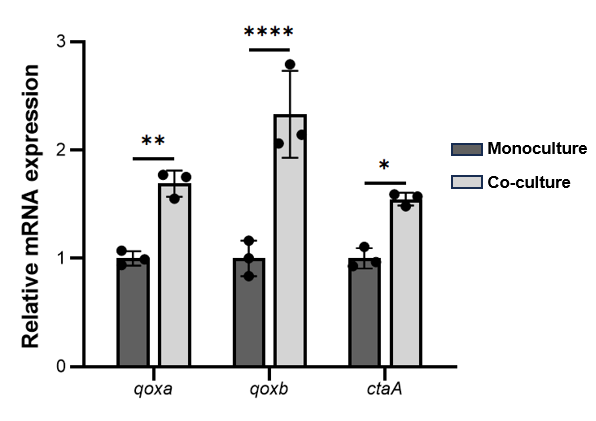


**Supplementary Figure S5. RT-qPCR results of *qoxA*, *qoxB* and *ctaA* genes in MRSA.**


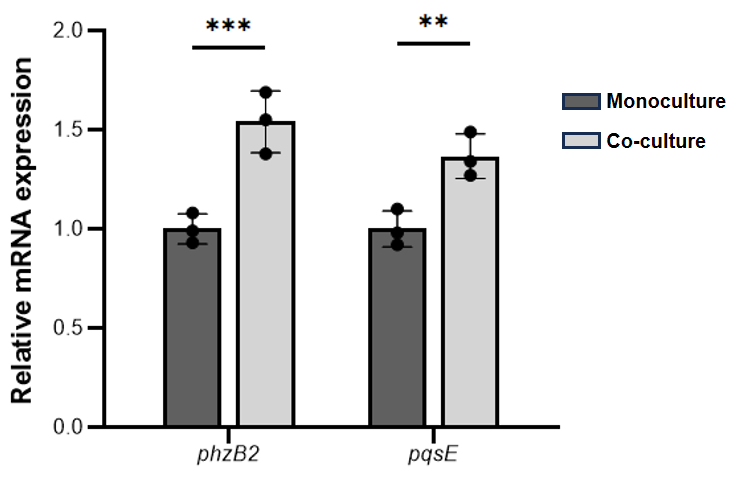


**Supplementary Figure S6. RT-qPCR results of *phzB2*, *pqsE* genes in PA.**

**Supplementary Table S1. Main bacterial strains, reagents, consumables, instruments, and software used in this study.**

| Item | Supplier / Manufacturer | Country |
| --- | --- | --- |
| Methicillin-resistant *Staphylococcus aureus*  ATCC BAA-1717 | Baosai Biotechnology | China |
| *Pseudomonas aeruginosa*  ATCC 15692 | Baosai Biotechnology | China |
| Luria–Bertani broth | Solarbio | China |
| Luria–Bertani agar | Solarbio | China |
| Mannitol salt agar | Hopebio | China |
| Pseudomonas CN agar | HuanKai Microbial | China |
| Transwell inserts | Labselect | China |
| Multi-mode microplate reader | Molecular Devices | USA |
| VITEK 2 automated system | bioMérieux | France |
| GP/GN antimicrobial susceptibility testing cards | bioMérieux | France |
| *Staphylococcus aureus*  ATCC 29213 | Baosai Biotechnology | China |
| *Pseudomonas aeruginosa* ATCC 27853 | Baosai Biotechnology | China |
| 0.22 µm syringe-driven filters | Jet Biofiltration | China |
| Phosphate-buffered saline | Servicebio | China |
| Crystal violet | Solarbio | China |
| Absolute ethanol | Biosharp | China |
| Bacterial viability staining kit | Solarbio | China |
| Laser scanning confocal microscope | Olympus | Japan |
| OlyVIA software, version 3.3 | Olympus | Japan |
| Imaris software, version 9.0.1 | Oxford Instruments | UK |
| ImageJ software | National Institutes of Health | USA |
| TRIzol reagent | Invitrogen | USA |
| Agilent 2100 Bioanalyzer | \| Agilent Technologies \| \| --- \| | USA |
| Ribo-Zero kit | Illumina | USA |
| NEBNext® Ultra™ II kit | New England Biolabs | USA |
| Illumina NovaSeq™ platform | Illumina | USA |
| DESeq2 software, version 1.46.0 | Bioconductor | USA |

**Supplementary Table S2. Reduction in MRSA viable counts after 48 h of co-culture with PA at different initial MRSA:PA ratios.**

| **Culture condition** | **Initial MRSA:PA ratio** | **Reduction vs MRSA monoculture (%)** |
| --- | --- | --- |
| MRSA monoculture | - | - |
| Co-culture | 1:1 | 27.1 |
| Co-culture | 1:100 | 17.7 |
| Co-culture | 100:1 | 9.2 |

**Supplementary Table S3. RT-qPCR primer sequences.**

| Name | Gene/Target | Sequence 5’→ 3’ |
| --- | --- | --- |
| gyrB F1 | gyrB | ACGGATAACGGACGTGGTATCCCA |
| gyrB R1 |  | GCCACCGCCGAATTTACCACCA |
| qoxA F | qoxA | CACCCA TTTGTC GTAGTCTT |
| qoxA R |  | GATTCCACA ATTAGGTGGTC |
| qoxB F | qoxB | GTTG TACT TGGC ATGT TCGC C |
| qoxB R |  | GGCA TTAT GGTG CATC TTAC C |
| ctaA F | ctaA | GCAGCTCCGATTAATGCTTG |
| ctaA R |  | CATGGTGCG TTG ATT CCA GA |
| rpsL F | rpsL | GCAAGCGCATGGTCGACAAGA |
| rpsL R |  | CGCTGTGCTCTTGCAGGTTGTGA |
| phzB2 F | phzB2 | GCGAGACGGTGGTCAAGTAT |
| phzB2 R |  | AATCCGGGAAGCATTTCAG |
| pqsE F | pqsE | GACATGGAGGCTTACCTGGA |
| pqsE R |  | CTCAGTTCGTCGAGGGATTC |
